# Persistent high levels of carcinoembryonic antigen after tumor resection are associated with poorer survival outcomes in patients with resected colon cancer

**DOI:** 10.1186/s12885-023-11126-4

**Published:** 2023-07-19

**Authors:** Wendy R. Muñoz-Montaño, Horacio N. López-Basave, Alison Castillo-Morales, Carolina Castillo-Morales, Karen Sánchez-Trejo, Rodrigo Catalán, Consuelo Díaz-Romero, Leonardo S. Lino-Silva, Andrea Maliachi-Díaz, Erika Ruiz-García, Marytere Herrera-Martínez, German Calderillo-Ruíz

**Affiliations:** 1 Department of Medical Oncology, Instituto Nacional de Cancerología, Av. San Fernando 22, Belisario Domínguez Secc. 16, Tlalpan, 14080 Ciudad de México, Mexico; 2 Mexican Agency for the Evaluation of Health Technologies, Mexico City, Mexico

**Keywords:** CEA values, Pre-operative and post-surgical, Colon cancer

## Abstract

**Background:**

Interindividual survival and recurrence rates in cases of locoregional colon cancer following surgical resection are highly variable. The aim of the present study was to determine whether elevated pre-operative and post-operative CEA values are useful prognostic biomarkers for patients with stage I-III colon cancer who underwent surgery with curative intent.

**Methods:**

We conducted a retrospective study in patients with histologically confirmed stage I-III primary colonic adenocarcinoma who underwent radical surgical resection at Mexico’s National Cancer Institute, between January 2008 and January 2020. We determined pre-operative and post-operative CEA and analyzed the association of scores with poorer survival outcomes in patients with resected colon cancer, considering overall survival (OS) and disease-free survival (DFS).

**Results:**

We included 640 patients with stage I-III colon cancer. Pre-operative CEA levels were in the normal range in 460 patients (group A) and above the reference value in the other 180. Of the latter, 134 presented normalized CEA levels after surgery, but 46 (group C) continued to show CEA levels above the reference values after surgery. Therefore, propensity score matching (PSM) was carried out to reduce the bias. Patients were adjusted at a 1:1:1 ratio with 46 in each group, to match the number in the smallest group. Median follow- up was 46.4 months (range, 4.9–147.4 months). Median DFS was significantly shorter in Group C: 55.5 months (95% CI 39.6–71.3) than in the other two groups [Group A: 77.1 months (95% CI 72.6–81.6). Group B: 75.7 months (95% CI 66.8–84.5) (*p*-value < 0.001)]. Overall survival was also significantly worse in group C [57.1 (95% CI 37.8–76.3) months] than in group A [82.8 (95% CI 78.6–86.9 months] and group B [87.1 (95% CI 79.6–94.5 months] (*p*-value = 0.002). To identify whether change in CEA levels operative and post-surgery was an independent prognostic factor for survival outcomes, a Cox proportional hazard model was applied. In multivariate analysis, change in CEA level was a statistically significant, independent prognostic factor for overall survival (*p*-value = 0.031).

**Conclusions:**

When assessed collectively, pre-operative and post-operative CEA values are useful biomarkers for predicting survival outcomes in patients with resected colon cancer. Prognoses are worse for patients with elevated pre-operative and post-surgical CEA values, but similar in patients with normal post-surgical values, regardless of their pre-surgery values.

## Introduction

Colon cancer (CC) is the fourth-most frequently diagnosed malignant neoplasia and the fifth-leading cause of cancer-related deaths worldwide [1]. The IDEA study showed that risk-based stratification is vital, and suggested that identifying a more appropriate prognostic biomarker is crucial for this malignancy [2]. Regarding this issue, clinical and pathological data including age, sex, tumor site, AJCC TNM stage, and the number of lymph node dissections (LND) have been shown to correlate with survival outcomes [3]. However, a feasible laboratory biomarker to appropriately assess risk of recurrence in CC is lacking.

Serum carcinoembryonic antigen (CEA) has been widely used as a biomarker in cases of CC [4]. Studies have demonstrated that higher pre-operative CEA concentrations correlate with worse outcomes in resectable CC. In the early 2000s, the Colon Working Group of the American Joint Committee on Cancer (AJCC) recommended including the serum CEA level at the moment of disease presentation in the conventional TNM staging of CC [5, 6]. Soon after, guidelines from the National Comprehensive Cancer Network (NCCN) [7], the European Society of Medical Oncology [8] (ESMO), and the American Society of Clinical Oncology (ASCO) [9] recommended routine measurement of pre-operative CEA levels before CC resection, for subsequent post-operative surveillance.

As has been reported previously, consistent elevation of CEA levels after tumor resection is a concerning sign for disease recurrence, so it has been a particularly useful biomarker during follow-up [10, 11]. Currently, measuring CEA before resection and every 3–6 months afterwards is recommended for patients with resected CC [12]. Numerous studies have demonstrated the usefulness of pre-operative CEA as a biomarker of prognosis in patients with CC who will undergo surgery. Other research suggests that measuring post-operative CEA also provides valuable prognostic information regarding disease recurrence [13, 14].

Evaluating the variability of pre-operative and post-operative CEA is another approach that has been tested. In this regard, outcomes of patients with a normalized CEA after surgery did not vary significantly when stratified according to pre-operative CEA values. In contrast, higher post-operative CEA values have been associated with worse outcomes, regardless of pre-operative levels [15].

High pre-operative CEA levels remained above reference values in approximately one-third of the patients with CC that underwent surgery with a curative intent. Alarmingly, this might indicate persistent disease and the need for further evaluation [16, 17]. With respect to the appropriate time after surgery to assess post-operative CEA, a retrospective analysis showed that measuringwithin a time frame of 21–100 days after resection is adequate to correlate CEA and disease-free survival (DFS) in patients with stage III CC, but the preferred time frame is during the first two months after surgery [18].

The aim of the present study was to determine whether elevated pre-operative and post-operative CEA values are useful prognostic biomarkers for patients with stage I-III colon cancer who underwent surgery with curative intent. Specifically, we set out to determine whether patients with elevated pre-operative CEA and non-normalized levels post-resection had a greater risk of recurrence than that of patients with normal pre-operative and normalized post-operative CEA values.

## Methods

**Fig. 1:**
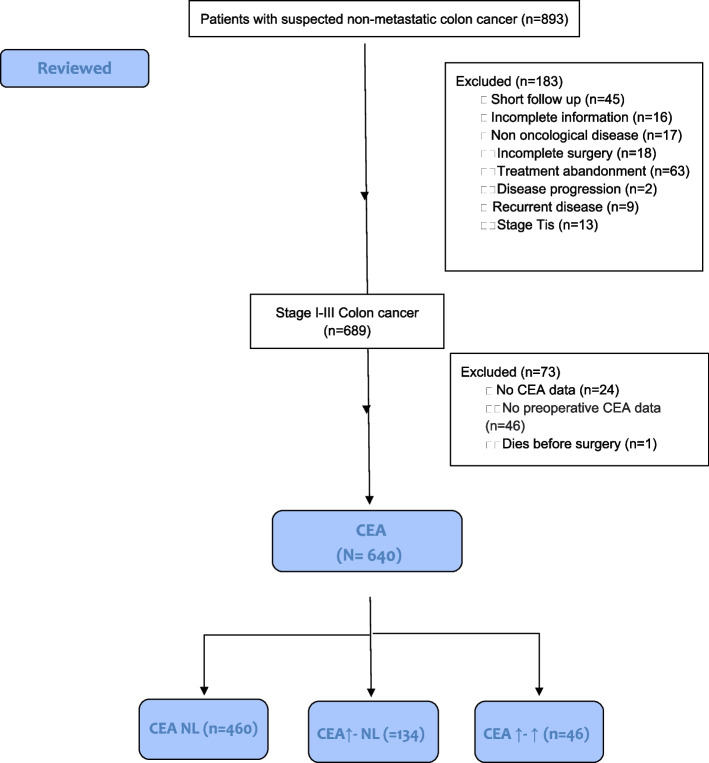
STROBE flow diagram

This study is based on a retrospective analysis of data of patients with colon cancer who underwent surgery with curative intent from January 2008 to January 2020 at Mexico’s National Cancer Institute (NCI). Patients were included if stage I-III colon adenocarcinoma was confirmed histologically and subsequently underwent radical surgery on the primary tumor. A database was elaborated by a multidisciplinary team. The variables included were pre-operative and post-operative CEA, family history of CC, synchronous malignancies, local excision, and palliative/adjuvant treatment, among others. Patients were excluded if ≥ 10% of the predetermined variables were not available in their medical records Fig. 1.

At the NCI, serum CEA levels are routinely measured using a microparticle enzyme immunoassay (ARCHITECT CEA Reagent Kit, ref. 7K68-27; Abbott, Wiesbaden, Germany). Reference values were predetermined at ≤ 5 ng/mL. In order to be included, measurements had to be performed before surgery and in the first three months after resection; that is, before the onset of any adjuvant treatment. According to the variability of CEA levels before and after surgery, patients were stratified into three groups: the first (group A) included patients with normal pre-operative and post-operative CEA (< 5 ng/mL); the second (group B) was comprised of patients with an elevated pre-operative CEA that was normalized after surgery; the third (group C) was made up of patients with pre-operative—and post-operative CEA levels above reference values.

Post-operative follow-up was performed every 3 months during the first 2 years after surgery, then every 6 months for up to 5 years. Patients were evaluated clinically by a medical oncologist who measured serum CEA levels on each visit. Abdominal, pelvic, and thoracic CT scans were performed every 6 months. Colonoscopies were performed 1 year after surgery and every 2 years thereafter.

The primary endpoints of this study were disease-free survival (DFS) and overall survival (OS). Recurrence was determined based on clinical and/or radiological signs of tumor development and histological confirmation. of Recurrence sites were categorized in three subgroups: local recurrence if at or near the anatomic site of the previously resected tumor; intra-abdominal recurrence; or distant recurrence (e.g., lymph node metastases). DFS was defined as the time between surgery and recurrence, death from any cause, or the final follow-up session. OS was defined as the time between surgery and death, or the date on which the patient was last confirmed to be alive.

For the statistical analysis, categorical variables were reported as counts and proportions. Comparisons among categorical variables were analyzed by an χ^2^ Fisher exact test. Continuous variables were reported as means and standard deviations (SD). Comparisons of means were evaluated using a T-test or ANOVA. Some of the patients’ characteristics showed statistically significant differences within the 3 CEA groups. Therefore, propensity score matching (PSM) was carried out to reduce bias. Patients were adjusted at a 1:1:1 ratio with 46 in each group to match the number in the smallest group. To obtain the matched cohort, a reference group was considered, then propensity-matched populations of the reference group *versus* the other two were obtained. Patients in these two groups were extracted if they had a common match to one in the reference group. DFS and OS analyses were conducted using the Kaplan–Meier method, and the log-rank test was used to determine any differences in survival among the subgroups. Multivariate analyses for prognostic factors were performed using a Cox proportional hazard model. Variables that were significant in the univariate analysis were included in the multivariate model. A *p*-value ≤ 0.05 was considered statistically significant. All statistical analyses were performed using SPSS software, version 26 (SPSS Inc., Chicago, IL, USA), and the programming language R version 4.0.5.

Individual patient information remained confidential throughout the protocol, and clinical management of patients was not influenced in any way by the results of our study. The project was approved by Institutional Review Board at Mexico’s National Cancer Institute with reference INCAN/CI/0687/2021, registered under number 2021/064. It was not necessary to obtain patients’ written informed consent due to the retrospective nature of the study. The project was conducted in accordance with the Helsinki Declaration and the Principles of Good Clinical Practice.

## Results

A total of 640 patients with stage I-III colon cancer were included in the final database for analysis. Pre-operative CEA levels were in the normal range in 460 patients, but above the reference value in the other 180. Of the latter, 134 presented normalized CEA levels after surgery, but 46 continued to show CEA levels above reference values after surgery. The median values of the pre-operative and post-operative CEA levels were 2.59 ng/mL (range, 0.28–3,068.5 ng/mL) and 1.95 ng/mL (range, 0.08–3,900.0 ng/mL), respectively.

As mentioned above, patients were stratified into 3 groups in accordance with their pre-operative—and post-operative CEA levels: 460 in group A, 134 in group B, and 46 in group C. There were no differences among the groups in terms of in age, gender, perineural invasion, tumor location, or post-operative complications. Advanced stage illness, presence of lymphatic/vascular invasion, and adjuvant treatment were most common in group 3, followed by group 2 Table 1. The general patient characteristics after PSM are shown in Table 2.

**Table 1:** General patient characteristics before PSM

			**CEA expression**			
Variables n (%)	**TOTAL (*n* = 640)**	**CEA NL (*n* = 460)**	**CEA ↑- NL (*n* = 134)**	**CEA ↑- ↑ (*n* = 46)**	***P*-value**	**Test SMD**
**Age group**					**0,129**	**0.223**
**< 40**	**91 (14.2)**	**74 (16.1)**	**12 (9.0)**	**5 (10.9)**		
**40–70**	**424 (66.3)**	**303 (65.9)**	**93 (69.4)**	**28 (60.9)**		
**> 70**	**125 (19.5)**	**83 (18.0)**	**29 (21.6)**	**13 (28.3)**		
**Gender**					**0,753**	**0.044**
**Female**	**319 (49.8)**	**225 (48.9)**	**70 (52.2)**	**24 (52.2)**		
**Male**	**321 (50.2)**	**235 (51.1)**	**64 (47.8)**	**22 (47.8)**		
**BMI in kg/m**^**2**^					**0,627**	**0.239**
**< 18.5**	**24 (3.8)**	**18 (3.9)**	**4 (3.0)**	**2 (4.3)**		
**18.5–24.9**	**315 (49.2)**	**229 (49.8)**	**61 (45.5)**	**25 (54.3)**		
**25–29.9**	**206 (32.2)**	**146 (31.7)**	**50 (37.3)**	**10 (21.7)**		
**≥ 30**	**95 (14.8)**	**67 (14.6)**	**19 (14.2)**	**9 (19.6)**		
**Pathological stage**					**0,064**	**0.45**
**I**	**75 (11.7)**	**63 (13.7)**	**8 (6.0)**	**4 (8.7)**		
**IIA**	**206 (32.2)**	**148 (32.2)**	**49 (36.6)**	**9 (19.6)**		
**IIB**	**55 (8.6)**	**37 (8.0)**	**15 (11.2)**	**3 (6.5)**		
**IIC**	**15 (2.3)**	**8 (1.7)**	**6 (4.5)**	**1 (2.2)**		
**IIIA**	**10 (1.6)**	**7 (1.5)**	**3 (2.2)**	**0 (0.0)**		
**IIIB**	**190 (29.7)**	**137 (29.8)**	**35 (26.1)**	**18 (39.1)**		
**IIIC**	**89 (13.9)**	**60 (13.0)**	**18 (13.4)**	**11 (23.9)**		
**Tumor differentiation**					***0,004***	**0.428**
**Well**	**121(18.9)**	**97 (21.1)**	**21 (15.7)**	**3 (6.5)**		
**Moderate**	**362 (56.6)**	**253 (55.0)**	**86 (64.2)**	**23 (50.0)**		
**Poor**	**157 (24.5)**	**110 (23.9)**	**27 (20.1)**	**20 (43.5)**		
**Tumor location**					**0,833**	**0.036**
**Right colon**	**322 (50.3)**	**228 (49.6)**	**70 (52.2)**	**24 (52.2)**		
**Left colon**	**318 (49.7)**	**232 (50.4)**	**64 (47.8)**	**22 (47.8)**		
**Lymphovascular invasion**					***0,029***	***0.266***
**No**	**421 (65.8)**	**309 (67.2)**	**90 (67.2)**	**22 (47.8)**		
**Yes**	**219 (34.2)**	**151 (32.8)**	**44 (32.8)**	**24 (52.2)**		
**Perineural invasion**					**0,13**	**0.219**
**No**	**523 (87.1)**	**376 (81.7)**	**114 (85.1)**	**33 (71.7)**		
**Yes**	**117 (18.3)**	**84 (18.3)**	**20 (14.9)**	**13 (28.3)**		
**Number of lymph nodes**					***0,001***	***0.29***
**< 12**	**95 (14.8)**	**82 (17.8)**	**6 (4.5)**	**7 (15.2)**		
**≥ 12**	**545 (85.2)**	**378 (82.2)**	**128 (95.5)**	**39 (84.8)**		
**Pathology T stage**					**0,085**	**0.297**
**T1**	**22 (3.4)**	**19 (4.1)**	**1 (0.7)**	**2 (4.3)**		
**T2**	**69 (10.8)**	**57 (12.4)**	**10 (7.5)**	**2 (4.3)**		
**T3**	**380 (59.4)**	**273 (59.3)**	**79 (59.0)**	**28 (60.9)**		
**T4**	**169 (26.4)**	**111 (24.1)**	**44 (32.8)**	**14 (30.4)**		
**Pathology N stage**					**0,223**	**0.26**
**N0**	**357 (55.8)**	**261 (56.7)**	**78 (58.2)**	**18 (39.1)**		
**N1**	**164 (25.6)**	**116 (25.2)**	**32 (23.9)**	**16 (34.8)**		
**N2**	**119 (18.6)**	**83 (18.0)**	**24 (17.9)**	**12 (26.1)**		
**Surgical margins/type of resection**					**0,188**	**0.193**
**R0**	**626 (97.8)**	**450 (97.8)**	**132 (98.5)**	**44 (95.7)**		
**R1**	**13 (2.0)**	**10 (2.2)**	**1 (0.7)**	**2 (4.3)**		
**R2**	**1 (0.2)**	**0 (0.0)**	**1 (0.7)**	**0 (0.0)**		
**Cancer obstruction**					***0,038***	***0.183***
**No**	**562 (87.8)**	**395 (85.9)**	**126 (94.0)**	**41 (89.1)**		
**Yes**	**78 (12.2)**	**65 (14.1)**	**8 (6.0)**	**5 (10.9)**		
**Cancer perforation**					**0,861**	**0.06**
**No**	**583 (91.1)**	**419 (91.1)**	**123 (91.8)**	**41 (89.1)**		
**Yes**	**57 (8.9)**	**41 (8.9)**	**11 (8.2)**	**5 (10.9)**		
**CCI**						
**0**	**180 (21.8)**	**147 (32.0)**	**26 (19.4)**	**7 (15.2)**	***0,007***	***0.36***
**1**	**152 (23.8)**	**110 (23.9)**	**27 (20.1)**	**15 (32.6)**		
**2**	**129 (20.2)**	**83 (18.0)**	**37 (27.6)**	**9 (19.6)**		
**≥ 3**	**179 (28.0)**	**120 (26.1)**	**44 (32.8)**	**15 (32.6)**		
**Postoperative complications**				**0,146**	**0.196**	
**No**	**551 (86.1)**	**400 (87.0)**	**109 (81.3)**	**42 (91.3)**		
**Yes**	**89 (13.9)**	**60 (13.0)**	**25 (18.7)**	**4 (8.7)**		
**Adjuvant chemotherapy**					**0,72**	**0.085**
**No**	**244 (38.1)**	**178 (38.7)**	**51 (38.1)**	**15 (32.6)**		
**Yes**	**396 (61.9)**	**282 (61.3)**	**83 (61.9)**	**31 (67.4)**		
**CEA Groups**					** < *0,001***	**NR**
**CEA NL**	**460(71.8)**	**460(100.0)**	**0(0.0)**	**0(0.0)**		
**CEA ↑- NL**	**134(20.9)**	**0(0.0)**	**134(100.0)**	**0(0.0)**		
**CEA ↑- ↑**	**46(7.1)**	**0(0.0)**	**0(0.0)**	**46(100.0)**		

*CEA* Carcinoembryonic antigen, *CCI* Charlson comorbidity index, *AJCC* American Joint Committee on Cancer, *PSM* Propensity score matching

**Table 2:** General patient characteristics after PSM

		**CEA Expression**				
**Variables n(%)**	**TOTAL (*n* = 138)**	**CEA NL (*n* = 46)**	**CEA ↑- NL (*n* = 46)**	**CEA ↑- ↑ (*n* = 46)**	***P* value**	**Test SMD**
**Age group**					**0,542**	**0.025**
**< 40**	**14 (10.1)**	**6 (13.0)**	**3 (6.5)**	**5 (10.9)**		
**40–70**	**84 (60.9)**	**24 (52.2)**	**32 (69.6)**	**28 (60.9)**		
**> 70**	**40 (29.0)**	**16 (34.8)**	**11 (23.9)**	**13 (28.3)**		
**Gender**					**0,686**	**0.116**
**Female**	**67 (48.6)**	**23 (50.0)**	**20 (43.5)**	**24 (52.2)**		
**Male**	**71 (51.4)**	**23 (50.0)**	**26 (56.5)**	**22 (47.8)**		
**BMI in kg/m**^**2**^					**0,369**	**0.346**
**< 18.5**	**8 (5.8)**	**3 (6.4)**	**3 (6.5)**	**2 (4.3)**		
**18.5–24.9**	**69 (50.0)**	**24 (52.2)**	**20 (43.5)**	**25 (54.3)**		
**25–29.9**	**40 (29.0)**	**11 (23.9)**	**19 (41.3)**	**10 (21.7)**		
**≥ 30**	**21 (15.2)**	**8 (17.4)**	**4 (8.7)**	**9 (19.6)**		
**Pathological stage**					**0,343**	**0.518**
**I**	**14 (10.1)**	**8 (17.4)**	**2 (4.3)**	**4 (8.7)**		
**IIA**	**30 (21.7)**	**6 (13.0)**	**15 (32.6)**	**9 (19.6)**		
**IIB**	**8 (5.8)**	**2 (4.3)**	**3 (6.5)**	**3 (6.5)**		
**IIC**	**4 (2.9)**	**1 (2.2)**	**2 (4.3)**	**1 (2.2)**		
**IIIA**	**4 (2.9)**	**2 (4.3)**	**2 (4.3)**	**0 (0.0)**		
**IIIB**	**52 (37.7)**	**17 (37.0)**	**17 (37.0)**	**18 (39.1)**		
**IIIC**	**26 (18.8)**	**10 (21.7)**	**5 (10.9)**	**11 (23.9)**		
**Tumor differentiation**				**0,303**	**0.304**	
**Well**	**17 (12.3)**	**5 (10.9)**	**9 (19.6)**	**3 (6.5)**		
**Moderate**	**70 (56.7)**	**23 (50.0)**	**24 (52.2)**	**23 (50.0)**		
**Poor**	**51 (37.0)**	**18 (39.1)**	**13 (28.3)**	**20 (43.5)**		
**Tumor location**					**0,575**	**0.146**
**Right colon**	**65 (47.1)**	**22 (47.8)**	**19 (41.3)**	**24 (52.2)**		
**Left colon**	**73 (52.9)**	**24 (52.2)**	**27 (58.7)**	**22 (47.8)**		
**Lymphovascular invasion**					**0,891**	**0.058**
**No**	**68 (49.3)**	**22 (47.8)**	**24 (52.2)**	**22 (47.8)**		
**Yes**	**70 (50.7)**	**24 (52.2)**	**22 (47.8)**	**24 (52.2)**		
**Perineural invasion**					**0,782**	**0.096**
**No**	**98 (71.0)**	**31 (67.4)**	**34 (73.9)**	**33 (71.7)**		
**Yes**	**40 (29.0)**	**15 (32.6)**	**12 (26.1)**	**13 (28.3)**		
**Number of lymph nodes**					**0,355**	**0.426**
**< 12**	**18 (13.0)**	**10 (21.7)**	**1 (2.2)**	**7 (15.2)**		
**≥ 12**	**120 (87.0)**	**36 (78.3)**	**45 (97.8)**	**39 (84.8)**		
**Pathology T stage**					**0,271**	**0.463**
**T1**	**4 (2.9)**	**2 (4.3)**	**0 (0.0)**	**2 (4.3)**		
**T2**	**14 (10.1)**	**8 (17.4)**	**4 (8.7)**	**2 (4.3)**		
**T3**	**74 (53.6)**	**20 (43.5)**	**26 (56.5)**	**28 (60.9)**		
**T4**	**46 (33.3)**	**16 (34.8)**	**16 (34.8)**	**14 (30.4)**		
**Pathology N stage**					**0,777**	**0.179**
**N0**	**57 (41.3)**	**17 (37.0)**	**22 (47.8)**	**18 (39.1)**		
**N1**	**49 (35.5)**	**17 (37.0)**	**16 (34.8)**	**16 (34.8)**		
**N2**	**32 (23.2)**	**12 (26.1)**	**8 (17.4)**	**12 (26.1)**		
**Surgical margins/type of resection**				**0,593**	**0.179**	
**R0**	**132 (95.7)**	**43 (93.5)**	**45 (97.8)**	**44 (95.7)**		
**R1**	**6 (4.3)**	**3 (6.5)**	**1 (2.2)**	**2 (4.3)**		
**R2**	**0 (0.0)**	**0 (0.0)**	**0 (0.0)**	**0 (0.0)**		
**Cancer obstruction**					**0,261**	**0.228**
**No**	**122 (88.4)**	**38 (82.6)**	**43 (93.5)**	**41 (89.1)**		
**Yes**	**16 (11.6)**	**8 (17.4)**	**3 (6.5)**	**5 (10.9)**		
**Cancer perforation**					**0,261**	**0.228**
**No**	**122 (88.4)**	**38 (82.6)**	**43 (93.5)**	**41 (89.1)**		
**Yes**	**16 (11.6)**	**8 (17.4)**	**3 (6.5)**	**5 (10.9)**		
**CCI**						
**0**	**26 (18.8)**	**7 (15.2)**	**12 (26.1)**	**7 (15.2)**	**0,555**	**0.318**
**v1**	**39 (28.3)**	**11 (23.9)**	**13 (28.3)**	**15 (32.6)**		
**2**	**24 (17.4)**	**7 (15.2)**	**8 (17.4)**	**9 (19.6)**		
**≥ 3**	**49 (35.5)**	**21 (45.7)**	**13 (28.3)**	**15 (32.6)**		
**Postoperative complications**				**0,754**	**0.093**	
**No**	**122 (88.4)**	**40 (87.0)**	**42 (91.3)**	**42 (91.3)**		
**Yes**	**16 (11.6)**	**6 (13.0)**	**4 (8.7)**	**4 (8.7)**		
**Adjuvant chemotherapy**					**0,968**	**0.031**
**No**	**46 (33.3)**	**15 (32.6)**	**16 (34.8)**	**15 (32.6)**		
**Yes**	**92 (66.7)**	**31 (67.4)**	**30 (65.2)**	**31 (67.4)**		
**CEA Groups**					** < *0,001***	**NaN**
CEA NL	**46(33.3)**	**46(100.0)**	**0(0.0)**	**0(0.0)**		
CEA ↑- NL	**46(33.3)**	**0(0.0)**	**46(100.0)**	**0(0.0)**		
CEA ↑- ↑	**46(33.3)**	**0(0.0)**	**0(0.0)**	**46(0.0)**		

*CEA* Carcinoembryonic antigen, *BMI* Body mass index, *CCI* Charlson comorbidity index. Values presented as number (%)

**Fig. 2:**
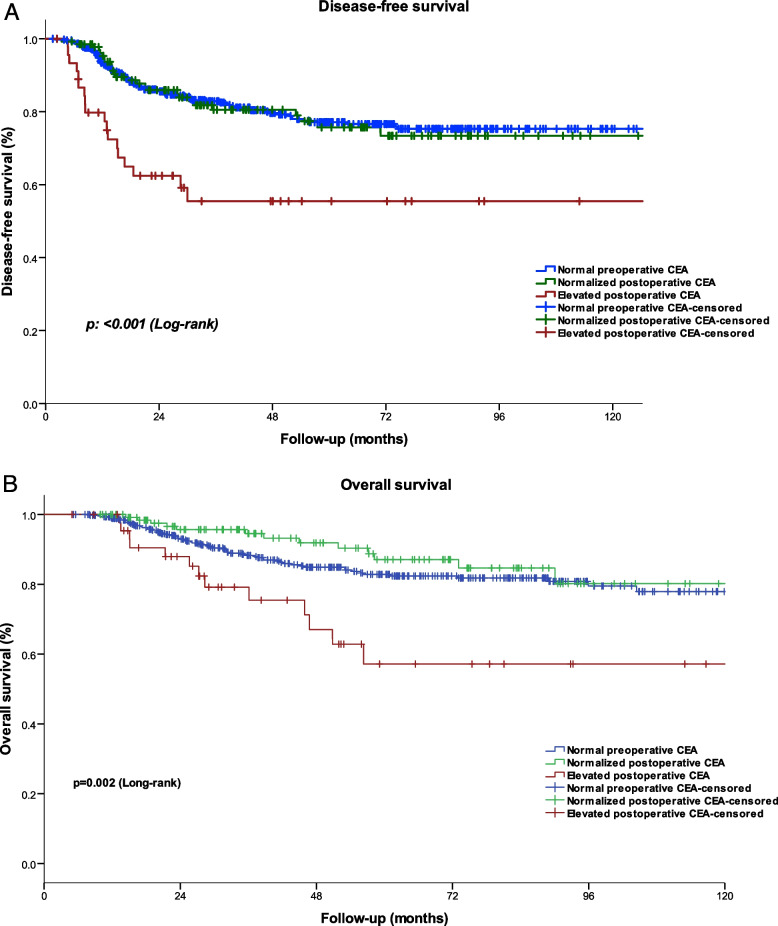
**A** Disease-free survival. **B** Overall survival

Median follow-up was 46.4 months (range, 4.9–147.4 months). Median DFS was significantly shorter in Group C at 55.5 months (95% CI 39.6–71.3) than in the other two groups [A: 77.1 months (95% CI 72.6–81.6)]; B: 75.7 months (95% CI 66.8–84.5) (*p*-value < 0.001)] Fig. 2A.

Overall survival was also significantly worse in group C [57.1 (95% CI 37.8–76.3) months] than in A [82.8 (95% CI 78.6–86.9 months] and B [87.1 (95% CI 79.6–94.5 months] (*p*-value = 0.002) Fig. 2B.

**Fig. 3:**
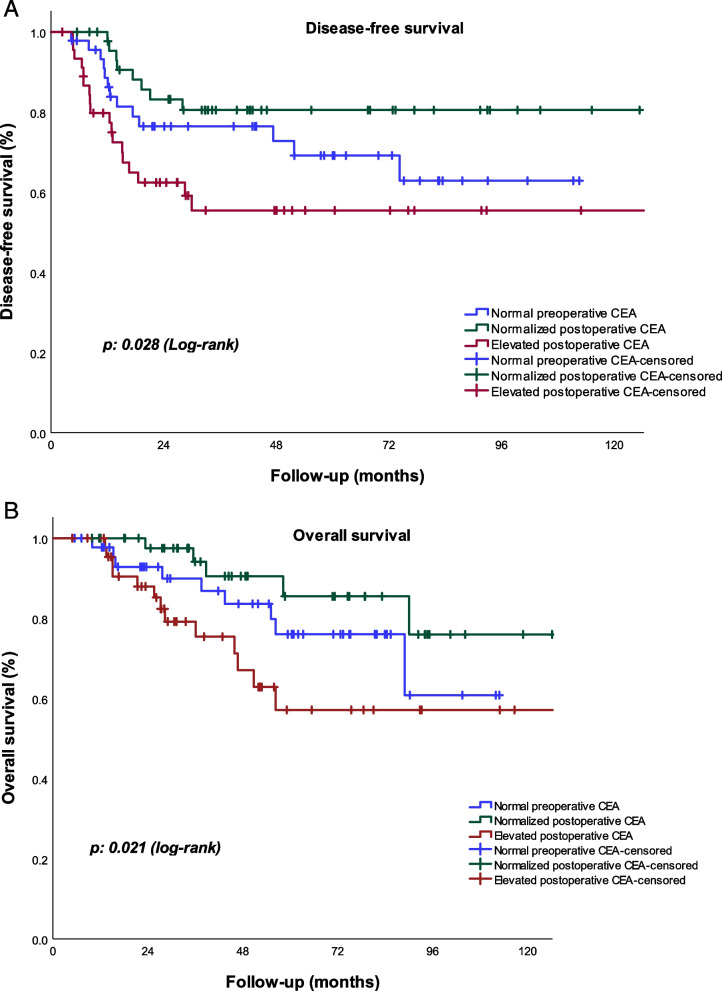
**A** Disease -free survival. **B** Overall survival

After PSM, we continued to identify differences in DFS and OS among the groups (*p* = 0.028 and *p* = 0.002, respectively) Fig. 3A and B.

To identify whether change in CEA levels pre-operative and post-surgery was an independent prognostic factor for survival outcomes, a Cox proportional hazard model was performed. The multivariate analysis showed that change in CEA levels was a statistically significant, independent prognostic factor for both overall survival (*p*-value = 0.041, Table 3) and disease-free survival (*p*-value = 0.029, Table 4).

**Table 3:** Univariate and multivariate models to predict OS in colon cancer patients with AJCC stage I-III after PSM

**5-year overall survival (OS)**					
	**Total (events)**	**Median (95% CI)**	***P*-value**	**HR (95% CI)**	***P*-value**
**Age (yrs.)**			**0.763**		
**< 65**	**88 (15)**	**76.1 (65.1–87.0)**			
**≥ 65**	**50 (12)**	**67.4 (49.7–85.0)**			
**Sex**			**0.181**		
**Female**	**67 (15)**	**66.3 (51.4–81.1)**			
**Male**	**71 (12)**	**79.0 (67.0–90.9)**			
**Pathological stage**			***0.047***		**.290**
**I**	**14 (0)**	**100**			
**II**	**42 (7)**	**78.6 (62.5–94.6)**			
**III**	**82 (20)**	**64.4 (50.8–77.9)**		**2.218 (0.507–9.714)**	
**Tumor differentiation**			***0.004***		**0.005**
**Well and moderate**	**87 (13)**	**81.9 (71.9–91.8)**			
**Poor**	**51 (14)**	**53.4 (32.2–74.5)**		**2.009 (1.234–3.270)**	
**Lymphovascular invasion**			**.077**		
**No**	**68 (10)**	**83.8 (73.4–94.4)**			
**Yes**	**70 (17)**	**60.5 (44.4–76.5)**			
**Perineural invasion**			**.068**		
**No**	**98 (18)**	**76.4 (66.0–86.8)**			
**Yes**	**40 (9)**	**63.1 (40.0–85.6)**			
**CCI**			**.455**		
**0**	**26 (3)**	**87.2 (73.9–100)**			
**≥ I**	**112 (24)**	**68.5 (56.9–80.0)**			
**CEA**			***0.021***		**0,041**
**NL**	**46 (9)**	**76.0 (61.1–90.8)**			
**↑- NL**	**46 (5)**	**85.5 (71.8–99.2)**			
**↑-↑**	**46 (13)**	**57.1 (37.8–76.3)**		**1.518 (0.926–2.489)**	
**Cancer obstruction**			**0.871**		
**No**	**122 (23)**	**74.5 (64.5–84.4)**			
**Yes**	**16 (4)**	**66.8 (39.7–93.8)**			
**Cancer perforation**			**0.914**		
**No**	**122 (25)**	**72.5 (71.5–73.4)**			
**Yes**	**16 (2)**	**82.5 (59.9–100)**			
**Number of lymph nodes**			**.645**		
**< 12**	**18 (5)**	**67.9 (44.5–91.2)**			
**≥ 12**	**120 (22)**	**74.3 (63.9–84.7)**			
**Pathology N stage**			***0.034***		**0.042**
**N negative**	**58 (7)**	**85.5 (74.3–96.6)**			
**N positive**	**80 (20)**	**63.5 (49.5–77.4)**		**2.448 (1.034–5.798)**	
**Surgical margins/type of resection**			**0.666**		
**R0**	**132 (25)**	**74.2 (64.5–83.8)**			
**R1/R2**	**6 (2)**	**55.6 (69.9–100)**			
**Adjuvant chemotherapy**					
**No**	**46 (33.3)**	**74.9(57.8–91.9)**	**0.841**		
**Yes**	**92 (66.7)**	**73.0(58.9–87.1)**			

*CEA* Carcinoembryonic antigen, *CCI* Charlson comorbidity index, *AJCC* American Joint Committee on Cancer, *PSM* Propensity score matching

**Table 4:** Univariate and multivariate models to predict DFS in colon cancer patients with AJCC stage I-III after PSM

**5-year disease-free survival (DFS)**					
		**HR (95% CI)**	***P*-value**	**HR (95% CI)**	***P*-value**
**Age (yrs.)**			**0.913**		
**< 65**	**88 (24)**	**69.3 (58.7–79.9)**			
**≥ 65**	**50 (15)**	**65.5 (49.8–81.1)**			
**Sex**			**0.249**		
**Female**	**67 (22)**	**61.9 (48.5–75.2)**			
**Male**	**71 (17)**	**73.3 (62.3–85.0)**			
**Pathological stage**			***0,049***		**0.411**
**I**	**14 (1)**	**91.7 (76.0–100)**			
**II**	**42 (10)**	**78.1 (65.3–90.8)**			
**III**	**82 (28)**	**58.5 (46.1–70.8)**		**1.491 (0.575–3.865)**	
**Tumor differentiation**			***0.006***		***0.006***
**Well and moderate**	**87 (19)**	**76.5 (66.8–86.1)**			
**Poor**	**51 (20)**	**50.1 (31.4–68.7)**		**1.682 (1.163–2.432)**	
**Lymphovascular invasion**			***0.039***		**0.806**
**No**	**68 (14)**	**78.6 (68.2–88.9)**			
**Yes**	**70 (25)**	**56.8 (42.9–70.7)**		**0.898 (0.382–2.114.)**	
**Perineural invasion**			**0.135**		
**No**	**98 (25)**	**71.7 (61.7–81.7)**			
**Yes**	**40 ( 14)**	**58.8 (41.5–76.0)**			
**Charlson comorbidity index**			**0.341**		
**0**	**26 (5)**	**80.4 (64.9–95.8)**			
**≥ I**	**112 (34)**	**61.0 (49.4–72.5)**			
**CEA**			***0.028***		***0.029***
**NL**	**46 (13)**	**69.2 (54.1–84.2)**			
**↑- NL**	**46 (8)**	**80.5 (68.3–92.6)**			
**↑-↑**	**46 (18)**	**55.5 (39.6–71.3)**		**1.387 (0.932–0.2.065)**	
**Cancer obstruction**			**.515**		
**No**	**122 (35)**	**67.2 (57.7–76.6)**			
**Yes**	**16 (4)**	**73.3 (50.9–95.6)**			
**Cancer perforation**			**0.992**		
**No**	**122 (35)**	**67.8 (58.6–77.0)**			
**Yes**	**16( 4)**	**71.8 (48.2–95.3)**			
**Number of lymph nodes**			**0.271**		
**< 12**	**18 (8)**	**51.9 (27.4–76.4)**			
**≥ 12**	**120 (31)**	**70.9 (61.8–79.9)**			
**Pathology N stage**			***0.026***		***0.028***
**N negative**	**58 (11)**	**81.9 (71.7–92.1)**			
**N positive**	**80 (28)**	**57.5 (44.9–70.0)**		**2.193 (1.091–4.407)**	
**Surgical margins/type of resection**			**0.201**		
**R0**	**132 (36)**	**68.9 (59.8–77.9)**			
**R1/R2**	**6 (3)**	**50.0 (10.0–89.9)**			
**Adjuvant chemotherapy**					
**No**	**46 (33.3)**	**75.5 (59.8–91.2)**	**0.841**		
**Yes**	**92 (66.7)**	**64.2(53.0–75.4)**			

*CEA* Carcinoembryonic antigen, *CCI* Charlson comorbidity index, *AJCC* American Joint Committee on Cancer, *PSM* Propensity score matching

## Discussion

Post-operative serum CEA is an accurate, cost-effective, widely available test that shows potential as a prognostic biomarker in stage II-III colon cancer. Findings regarding the prognostic value of post-operative CEA could provide evidence to guide individualized adjuvant treatment of stage II-III colon cancer. For example, patients with high CEA after resection with no other known risk factors could benefit from more intensive adjuvant therapy, while those with lower post-operative serum CEA values could avoid aggressive treatments and their potential undesirable effects [18]. However, additional prospective studies are needed to verify the role of CEA in determining survival outcomes and its usefulness for guiding decisions regarding adjuvant treatment.

In this study, we analyzed the prognostic value of changes in CEA levels pre-operative and post-surgery in CC patients who underwent radical surgery with curative intent. We identified an association between persistent high CEA levels after surgery and worse survival outcomes. In addition, such features as bowel obstruction or perforation, advanced-stage cancer, and the presence of lymphatic, vascular, or perineural invasion were associated with poor outcomes. These results proved to be statistically significant in the multivariate analysis after PSM, so they can be considered robust. Many studies have explored the role of serum CEA as a prognostic indicator in colon cancer. Observational studies have found that pre-operative CEA level is a significant indicator for recurrence and survival, while others have determined that post-operative CEA is an independent prognostic factor [8, 12, 14, 18]. Population-based studies with large cohorts have reported that pre-operative CEA level is only a poor independent prognostic factor [14, 17], but they were based on insufficient CEA data, failed to adjust for clinical features, and included patients with palliative surgery. Other studies do not report any significant CEA findings with respect to oncological outcomes [14]. Konishi et al [15] reported similar results to ours; indeed, their research suggests that patients with elevated post-operative CEA present an increased risk for recurrence compared to those with CEA values that returned to reference levels post-surgery.

Earlier studies demonstrated that elevated pre-operative CEA levels are associated with advanced-stage cancer, higher recurrence rates, and worse survival outcomes. Furthermore, high post-operative CEA levels are strongly associated with residual disease and/or distant metastases [16, 17].

,Iit is important to determine the prognoses and risk of recurrence of all CC patients, so there is an urgent need to identify biomarkers for this purpose [14, 17, 18]. Measuring serum CEA is simple, fast, inexpensive, and reliable, so it is a widely used biomarker in cases of CC [18]. Our study confirms that variability in CEA levels pre-operative and post-surgery has clinical and prognostic implications that allow us to appropriately identify patients with a higher risk of recurrence who may benefit from aggressive adjuvant treatment.

Our study has some limitations: first, its retrospective nature is an intrinsic limitation; second, it was a single-center project; and, third, we did not aim to establish a definitive cut-off value for elevated CEA, but only used a predetermined value of 5 ng/mL. Despite these features, our study had the strength of using propensity score matching to evaluate the prognostic value of CEA levels and overcome the confounding bias among the 3 study groups. Finally, we evaluated CEA levels pre-operative and post-surgery to assess the direction of variability in these patients.

## Conclusion

When assessed in conjunction, pre-operative- and post-operative CEA values are useful biomarkers for predicting survival outcomes in patients with resected CC. Prognoses are worse for patients with elevated pre-operative and post-surgical CEA values, but similar in patients with normal post-surgical values, regardless of their pre-operative -surgery levels.

## Data Availability

The original contributions presented in the study are included in the article.

## Abbreviations

CC: Colon cancer
LND: Lymph node dissection
CEA: Carcinoembryonic antigen
OS: Overall survival
DFS: Disease-free survival
KKS: Kallikrein-kinin system
RT: Radiotherapy
CRT: Chemoradiotherapy
